# Small Bowel Cocoon: A Distinct Disease with a New Developmental Etiology

**DOI:** 10.1155/2011/940515

**Published:** 2011-10-18

**Authors:** Ibrahim E. Bassiouny, Tariq O. Abbas

**Affiliations:** Pediatric Surgery Department, Hamad General Hospital, Doha 3050, Qatar

## Abstract

We have treated two patients with small bowel (abdominal) cocoon who presented with acute and partial small bowel obstruction associated with an abdominal mass. Neither had a history of previous surgery, peritonitis, or prolonged drug therapy. The distinct features of this disease are illustrated. A developmental etiology and a new nomenclature for this disease are proposed, along with a review of the literature.

## 1. Introduction

Small bowel (abdominal) cocoon is a rare disease of the peritoneum, of obscure origin, seen mostly in children and adolescents. It is characterized by the conglomeration of part of or the entire small bowel encased in a dense, white, fibrous membrane resulting in mechanical bowel obstruction. Frequently, a long-standing incomplete small bowel obstruction precedes the acute episode. No abnormality of intestinal rotation is associated with this entity and surgery can lead to complete recovery. 

We report our experience with two such children.

## 2. Case Reports

### 2.1. Patient 1

A 7-year-old Iranian boy presented with a 12-hour history of crampy abdominal pain and bilious vomiting. Beginning at age 3 years, he had experienced episodic colicky abdominal pain with vomiting, which subsided spontaneously.

Physical examination revealed a temperature of 36.6°C, a blood pressure of 110/70 mm Hg, and a pulse of 90 beats per minute. His abdomen was mildly distended with exaggerated bowel sounds. A soft, multiloculated, mobile, tender mass, 5 cm in diameter, could be palpated in the left upper abdominal quadrant. Laboratory findings were normal. 

**Figure 1:**
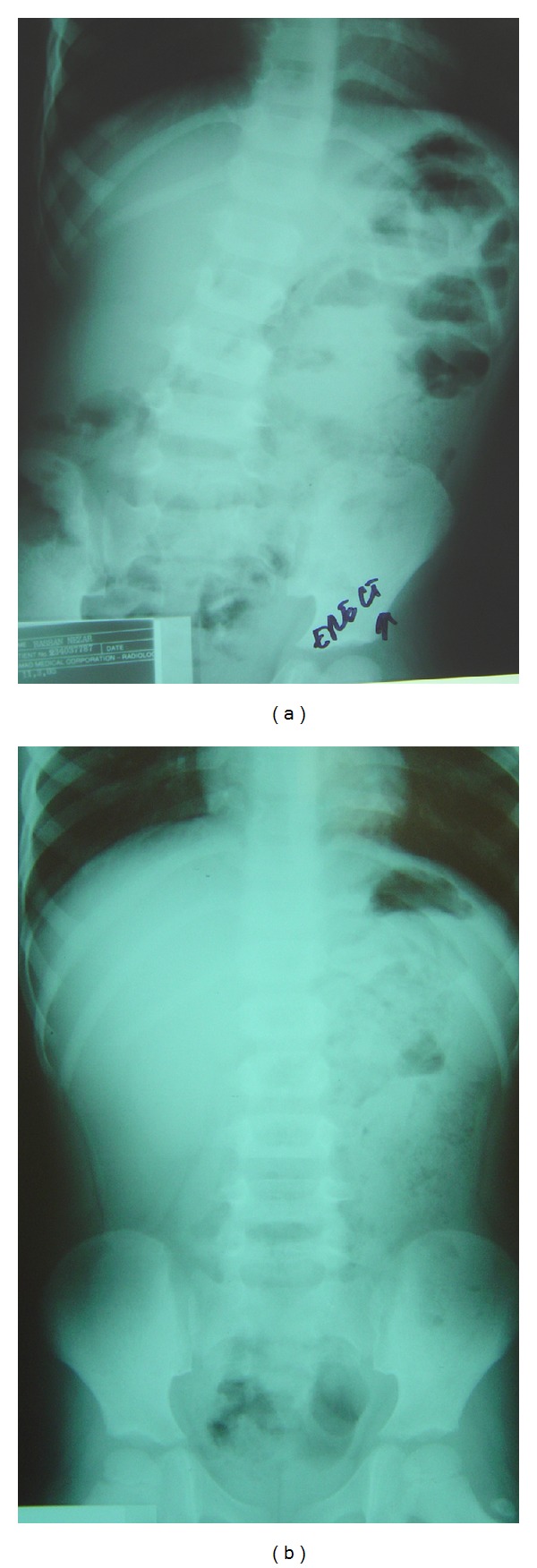
Plain X-ray of the abdomen of patient 1, showing (a) dilatation and conglomeration of the proximal small bowel in the left upper quadrant of the abdomen, (b) the fixed clusters of small bowel maintained a constant position.

Plain X-rays of the abdomen showed dilatation and conglomeration of the proximal small bowel in the left upper quadrant of the abdomen. Later, we found fixed clusters of small bowel maintained in a constant position (Figure 1). Contrast enema showed normal results.

**Figure 2:**
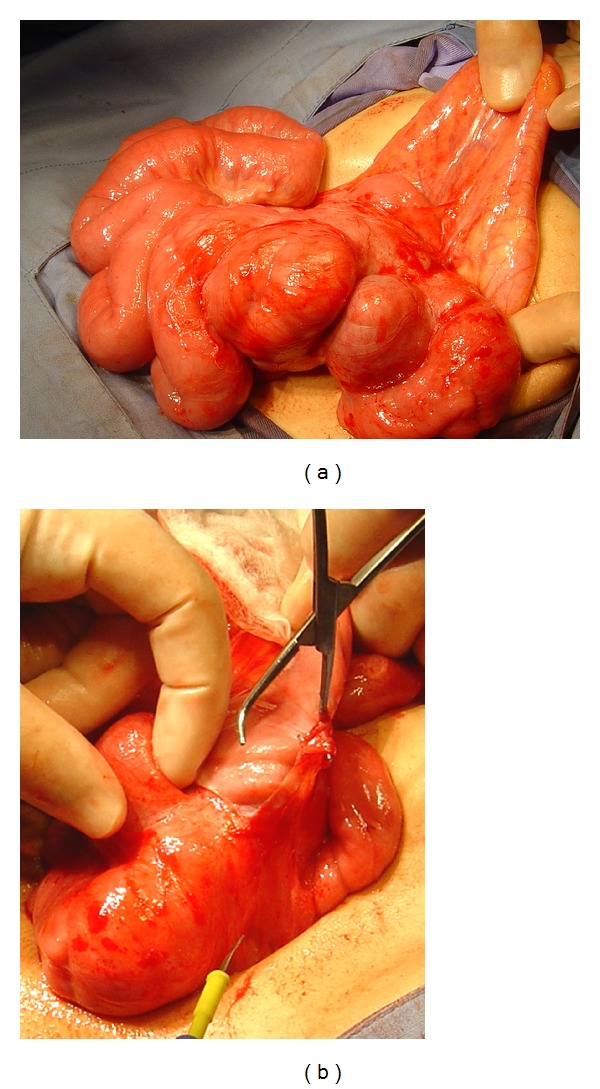
(a) Operative findings in patient 1, showing a foreshortened small bowel encased in a firm, rigid, fibrous membrane firmly adherent to the bowel. (b) View following incision of the membrane, revealing compressed, coiled, distended small bowel loops with interloop adhesions.

At surgery, we observed a foreshortened, distended, small bowel, encapsulated within a thick fibrous membrane adherent to the bowel serosal layer and fused to its mesentery. There was no evidence of malrotation. Incision of the fibrous membrane along the antimesenteric border of the small bowel revealed compressed, convoluted loops of small bowel under considerable tension with adhesions between the bowel loops. The sac and adhesions were easy to peel off by blunt dissection without affecting bowel viability. This resulted in normal-appearing bowel loops of normal length (Figure 2).

Histopathological examination of the membrane showed peritoneum with chronic inflammatory cells and fibrosis.

Postoperatively, the patient's symptoms were relieved, and he was discharged on the sixth day. He remains asymptomatic during followup.

### 2.2. Patient 2

A 12-year-old Egyptian girl was admitted due to severe, colicky abdominal pain, vomiting, and constipation of 24-hour duration. She had a 4-year history of intermittent abdominal distention, crampy abdominal pain, and recurrent episodes of vomiting, with normal bowel motion. 

Physical examination on admission revealed a well-developed girl with a temperature of 37°C, a blood pressure of 110/75 mm Hg, and a pulse of 95 beats per minute. Her abdomen was moderately distended with mild diffuse tenderness but without rigidity. A soft, mobile mass was palpable in the suprapubic area.

Abdominal X-rays showed distended loops of the small bowel with an obstructive pattern. Abdominal ultrasonography showed distended bowel loops with no definite mass.

**Figure 3:**
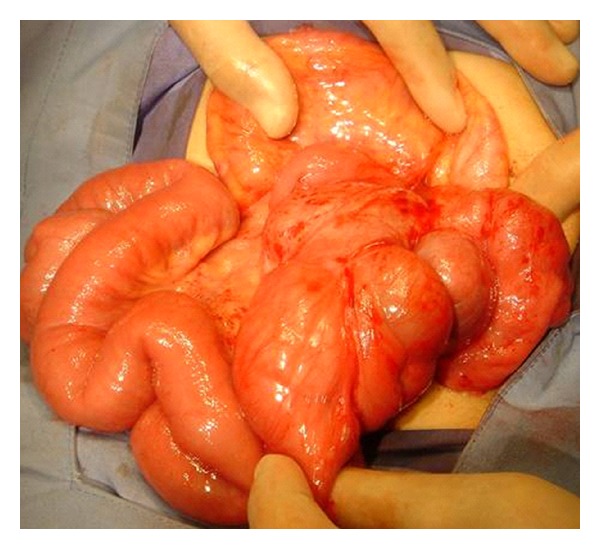
Patient 2; small bowel loops encased by a sac.

Following a presumptive diagnosis of partial small bowel obstruction, we performed an exploratory laparotomy. The small bowel was markedly shortened, distended, coiled in a concertina-like fashion and encased within a thick fibrous membrane. No bowel malrotation was detected. The fibrous sac was partially excised, and the interloop intestinal adhesions were lysed, resulting in a normal length of viable bowel (Figure 3).

Histologic examination of the encased membrane yielded results consistent with peritoneum with chronic inflammation and fibrosis. The patient's postoperative recovery was uneventful.

## 3. Discussion

Small bowel (abdominal) cocoon and peritoneal encapsulation are rare diseases of the peritoneum. Although these terms have been erroneously used interchangeably [7–11], they are two distinct pathological disorders. 

The term “abdominal cocoon” was first applied to a group of adolescent girls who presented, within 1–6 years of menarche, with small bowel obstruction due to “plastic adhesions” and a fibrous membrane encasing the small bowel totally or partially, in the manner of a cocoon [1]. This condition has predominantly tropical and subtropical distribution [10], with fewer than 50 patients reported to date [10, 12]. 

The etiology of small bowel (abdominal) cocoon remains obscure. It is not associated with any abnormality of intestinal rotation. Due to its initial predominance in adolescent girls, it was originally suggested that this condition may be due to retrograde menstruation, together with subclinical viral primary peritonitis, resulting in the development of an encapsulating membrane on the small bowel [1]. Alternatively, this condition may be a sequel to retrograde peritonitis via fallopian tubes caused by an endemic microorganism that has a predilection for the genital tract [6].

Small bowel (abdominal) cocoon is manifested as an acute or subacute mechanical small bowel obstruction, mostly associated with an abdominal lump [1–6, 8]. This condition is frequently preceded by long-standing incomplete intestinal obstruction, which manifests as intermittent non-specific abdominal pain, discomfort, and vomiting.

Peritoneal encapsulation was first described in 1868 [13]. Since then, only about 40 cases have been reported [4, 5, 14, 15]. This condition is characterized by the small bowel lying behind a thin, accessory but otherwise normal peritoneal membrane without being adherent to its serosal layer [16]. This membrane is attached to the ascending and descending colon laterally, to the transverse mesocolon cranially, and to the posterior parietal peritoneum caudally. The accessory peritoneal sac is derived from the yolk sac [3, 15, 17–20]. Paraduodenal hernia may be part of this phenomenon [17, 18]. The relative position of the viscera is normal, as is the length of the small bowel.

Clinically, most patients have been identified incidentally during unrelated surgery or at autopsy [3, 21] and most late in life as the condition is largely asymptomatic. Only 4 cases have been reported in children [3, 6, 8, 9, 21, 22]. Few patients present with bowel obstruction [6, 22]. When encountered incidentally during surgery, the membrane can be easily excised. 

Abdominal cocoon and peritoneal encapsulation are regarded as two sides of the same coin [7]. Depending on a patient's age and presentation, a bowel obstruction in a child, with or without a lump, is called an abdominal cocoon. In elderly individuals, when detected accidently during unrelated surgery, it is considered peritoneal encapsulation. 

When peritoneal encapsulation accompanies a chronic inflammatory process, it may result in a cocoon-like appearance or sclerosing encapsulation peritonitis [8]. However, most patients with abdominal cocoon are children or adolescents with no history of chronic disease. 

To resolve this problem, we propose that abdominal cocoon be renamed small bowel cocoon as it is more descriptive and meaningful etiologically and clinically. Small bowel cocoon should be regarded as a developmental anomaly of the peritoneum, resulting from the encapsulation of the small bowel within an accessory semirigid peritoneal sac derived from the yolk sac during small bowel return from the extracoelomic cavity to the abdominal cavity itself during the twelfth week of gestation. Enclosure within this semirigid membrane will block the growth of the small bowel, both in length and circumference, resulting in foreshortening of small bowel length. That is, the length of the small bowel as measured along its antimesenteric border is about 20% of the normal expected length according to a patient's age. This enclosure also results in coiling and compression of the bowel loops, the development of interloop adhesions, and the progressive firm adherence of the sac to the bowel serosal layer and the root of mesentery. Over time, a mobile mass made of bowel conglomeration will develop. The most striking feature of small bowel cocoon seen at surgery supports this hypothesis. 

Clinically, small bowel cocoon presents early in life with symptoms of long-standing partial bowel obstruction prior to acute obstruction of the small bowel, which also supports our hypothesis.

Small bowel cocoon has been reported in boys [7, our case] and in girls less than eight years of age, well before menarche [2, 7] (Table 1). There is no histological evidence of fungi, parasites, bacteria, viral infection [1, 4, 16] or history of drug use [23]. 

It is difficult to establish a diagnosis of small bowel cocoon preoperatively. This condition is mostly diagnosed intraoperatively. However, symptoms of intestinal obstruction accompanied by an abdominal lump and a serpentine configuration of the dilated small bowel within a cocoon may suggest a preoperative diagnosis of small bowel cocoon [6]. Contrast follow through, however, is not advisable in patients with acute bowel obstruction. Ultrasonography showing an abdominal mass composed of distended bowel containing air and solid matter without fluid may suggest small bowel cocoon, as may computerized tomography evidence of membrane changes accompanied by evidence of a fixed cluster of intestinal loops [22]. 

Surgery for small bowel cocoon consists of an incision in the fibrous capsule encasing the compressed small bowel and separation of the flimsy adhesion between bowel loops by blunt dissection. This will result in normal small bowel length. Bowel resection is unnecessary. 

## 4. Conclusion

We suggest using the term small bowel cocoon to describe small bowel obstruction associated with an abdominal mass in children and adolescents of both sexes with no obvious cause. 

We think that small bowel cocoon is a developmental disease due to continual growth of the small bowel loops within a semirigid encased accessory peritoneal membrane resulting in long-standing symptoms of partial bowel obstruction and abdominal mass before the onset of an acute obstructive episode. A better awareness of the clinical features of this condition may facilitate preoperative diagnosis and intraoperative identification, thus preventing inadvertent bowel damage and unnecessary bowel resection at laparotomy.

## Figures and Tables

**Table 1:** Summary of reported cases of abdominal cocoon in the literature.

	No. of Cases	Age (yr)	Sex	No. with acute or subacute SB obstruction	No. with abdominal mass
Foo et al. [1]	10	13–18	F	8	6
Rao et al. [2]	1	4	F	1	1
Sayfan et al. [3]	1	12	F	1	—
Marinho and Adelusi [4]	1	17	F	—	1
Awasthi et al. [5]	1	16	F	1	—
Sieck et al. [6]	1	14	F	1	—
Sahoo et al. [7]	4	6–8	3 M; 1 F	4	3
Mordehai et al. [8]	2	14–15	F	2	2
